# Time as a Category in Survivors’ Reports About Child Sexual Abuse: An Explorative Approach to Lifetime Abuse

**DOI:** 10.1177/08862605241264525

**Published:** 2024-09-10

**Authors:** Sabine Andresen

**Affiliations:** 1 Johann Wolfgang Goethe-University, Frankfurt am Main, Germany

**Keywords:** time, lifetime abuse, Independent Inquiry into Child Sexual Abuse in Germany, child sexual abuse, testimonies of survivors, bearing witness

## Abstract

Since the 1990s, cases of serious violence and abuse, particularly sexual abuse in educational and social institutions of the Catholic Church, have been reported in numerous countries, including Ireland, the United States, and Australia. In many countries, commissions have been set up to investigate the widespread cases of abuse that could not be prosecuted under criminal law. The testimonies of survivors and other witnesses are used by the commissions of inquiry in their work. Since 2016 the Independent Inquiry into Child Sexual Abuse in Germany has collected more than 2,500 reports from survivors of child sexual abuse. Forty-four testimonies were analyzed in a study seeking to understand more about lifetime abuse. In an explorative analysis, aspects of time as a key category in the reports were identified using the structured content analysis method. The results highlight the importance of time in the reports and during all phases of the survivors’ lives. Overall, four themes are relevant: (a) the identification of turning points, (b) time to make sense of what happened, (c) the practice of waiting, and (d) time-bound experiences of testifying.

In several countries, including Australia, Canada, the United Kingdom, Germany, Ireland, Israel, Sweden, and Switzerland, commissions have been established by governments and non-governmental organizations to investigate human rights violations in the recent past against children and young people (for an overview see Sköld & Swain, 2015; Wright, 2021). Published reports containing testimonies by survivors of child sexual abuse and other forms of violence and humiliation were a central starting point for the conception and establishment of these commissions. Originally, the accounts from survivors and other witnesses mainly concerned the Catholic Church, with clergy accused of being perpetrators and church leaders of protecting them (O’Donnell et al., 2021; Katsch, 2020). This was followed by accounts from survivors from the world of sport, well-known boarding schools, clinics, and clergy from the Protestant Church, as well as reports of child sexual abuse in other religious contexts. The main worldwide focus was on institutions and out-of-home care (Sköld, 2013).

Since the 1970s, survivors of child sexual abuse by family members, especially female survivors, have testified about their suffering, and some independent commissions, such as those in Germany and Israel, also accept reports of familial sexual abuse (Andresen, 2023).

The commissions use different methods and sources to obtain knowledge about the extent of violence and abuse, the perpetrators, the failure to help survivors, and about cover-ups. One method is based on the concept of bearing witness, especially in relation to testimony from survivors, and this is the approach employed by the Independent Inquiry into Child Sexual Abuse in Germany.

For research into lifetime abuse, the Inquiry offers an opportunity to gain new knowledge. Survivors also talk about sexual abuse and, in many cases, other forms of violence experienced in later phases of their lives. Many reports to the Inquiry come from people in later life. They want to report their experiences of child sexual abuse even when they are 50, 60, or 70 years old. This is why their testimonies, in particular, could offer new insights into a variety of medical, social, and/or financial aspects of lifetime abuse, including the impact on health and social relations and the risk of social isolation and socioeconomic consequences. The Inquiry in Germany as well as commissions in other countries have been able to access survivors’ court records documenting violence and victimization during adulthood.

This article explores facets of lifetime abuse that have become visible through analysis of testimonies from the Inquiry in Germany. In an exploratory study, 44 survivors’ testimonies were analyzed using qualitative content analysis with a focus on time. The focus is on the significance of time—past, present, and future—in the sense of the thematization of changes during the individual’s childhood and life course, including the return of traumatic memories, or even healing. The reports provide information about child sexual abuse in the past and about the survivors’ experiences in the present. They often also speak about their personal future and the future of child protection in society.

## Background

### The Independent Inquiry Into Child Sexual Abuse in Germany

Since the 1990s, reports on cases of serious violence and abuse (Wright, 2017, 2021) have been published in various countries, including Ireland, the USA, and Australia. Such international reports and findings from official inquiries are still relevant today. Yet findings that sexual abuse, the protection of perpetrators, and active cover-ups have taken place worldwide and on a vast scale under the umbrella of the Catholic Church, and the fact that the Church has also been part of massive human rights crimes against Indigenous children, as in Canada, and is only gradually facing up to its responsibility, have contributed to a kind of turning point (Nagy, 2013).

As in many other countries (see Salter, 2016), sexual abuse of children within families had already been publicly discussed in Germany in the 1970s and 1980s (Kavemann & Lohstöter, 1984). These discussions were initiated by feminists, survivors, and empowerment activists (Salter, 2016). However, at that time and against a background of associated criticism of bourgeois families and patriarchal power, the phenomenon of child sexual abuse in Germany did not receive the public attention it did in 2010. Here, “2010” is considered to symbolize an important turning point toward a political examination of child sexual abuse, civil society responsibility, and broad media coverage. In Germany, some changes have since been made both at the national level and in individual federal states.

In addition to the political response from the national government that led in 2010 to the setting up of a Round Table on Child Sexual Abuse,^1^ the position of an Independent Commissioner for Child Sexual Abuse Issues in Germany was established.^2^ Furthermore, a victims’ council (German Survivors’ Board^3^) and the Independent Inquiry into Child Sexual Abuse^4^ were set up in 2016, and since 2018 there has been a National Council against Child Sexual Abuse.^5^

The German Independent Inquiry into Child Sexual Abuse has been undertaking work based on survivors’ testimonies. The establishment of the Inquiry formed part of a global approach to addressing injustice perpetrated against children under the umbrella of the state, churches, health services, and education. Objectives include acknowledgment of the injustice, but also the production of collective knowledge about violence and abuse against children, who are significantly dependent, and about the role of bystanders in society who failed to protect them (Wright, 2017).

The Inquiry in Germany, similar to the inquiry in the United Kingdom, focuses primarily on child sexual abuse.^6^ A unique aspect of the German Inquiry is its mandate, which includes the investigation of two political systems, the German Democratic Republic (GDR) and the Federal Republic of Germany (FRG), from 1949 to the present. The main methods it employs to achieve this task are conducting confidential hearings with survivors and other witnesses; creating a database and clarifying how archiving will take place; organizing public hearings and conferences which are streamed live and can be viewed online afterward; holding “workshop discussions” and documenting them; and publishing case studies, statements, and recommendations (Independent Inquiry into Child Sexual Abuse in Germany [IICSAG], 2019).

Serving in a voluntary capacity, the Inquiry’s members (between five and seven) come from a range of disciplines and professions, for example, education, sexology and psychotherapy, sociology, law, and social psychology. They are supported by a team of professional assistants and receive critical input from survivors of child sexual abuse, especially from members of the German Survivors’ Board and from Germany’s Independent Commissioner for Child Sexual Abuse Issues.

People ranging from 16 years old to over 90 have contacted the Inquiry and shared their stories. Since May 2016, more than 2,500 people have registered to report their experiences, close to 1,900 confidential hearings have been held, and over 700 submissions of written testimony have been received (IICSAG, 2023). Women in general, and especially those aged between 51 and 60 make up the largest group. Statistics from 2023 show that 67% of 2,038 testimonies concerned sexual abuse within the family.^7^

The duration of the Inquiry has been extended again until 2025. The aim in Germany is to secure the subjective right of survivors to reappraisal, healing, and reparation.

### The Bearing Witness Approach

Bearing witness can take different forms, one of which is when the witness produces truth and speaks about facts as an eyewitness. “Survivor witnesses” bear witness to something life-threatening, often violence (Schmidt, 2017). They document how they experienced fear of death, war, or fleeing danger. As survivor witnesses, they also bear witness to those who have died. Witnesses of faith who report on an “inner truth” also have a long tradition (Schmidt, 2017). Thus witnesses are “bearers of knowledge” and a person bearing witness can be ascribed the character of a source of knowledge. Witnessing addresses the “interplay” of forgetting and remembering and, as a social knowledge practice, is dependent on a resonance space and on listeners.

In the concept of bearing witness to child sexual abuse, the category of time is central. Testimony is usually given about something that has happened, something that occurred in the recent or more distant past. However, through testimony, it is also possible to show that past events, especially if they deal with violence and abuse, are significant for the present and the future. A person who gives testimony is dependent on a space, an audience, which receives this testimony and is prepared to listen and to believe.

This is central to the commissions that build on the transitional justice approach and investigate violence against children (Aptel & Ladisch, 2011). Victims of sexual abuse and children, in their precarious position within the generational order, in families, at school, and in society at large, have in countless cases not been listened to when they have tried to disclose their experiences to someone. They have often not been believed and they have not been helped (Schaumann et al., 2022).

Consequently, in addition to the past perpetrator-victim dynamic, a “third” element comes into play in the investigation of child sexual abuse: Namely, the role of bystanders in the child’s environment. This complex dynamic is also to be explored through the process of bearing witness: “There are people who witness something over time, but who then do nothing, for whatever reason” (IICSAG, 2019, p. 105). This sentence from a survivor’s report corresponds to a pattern in which the complex dynamics become apparent.

In many cases, the third parties or bystanders from childhood remain relevant in adulthood. If a survivor of child sexual abuse within the family is speaking about the grandfather who abused her for years, the responses of siblings, parents, and other relatives can also differ extremely widely. Some believe the survivor’s testimony, some are encouraged to also disclose their experiences of abuse, and others may resist or accuse the witnesses of lying. Similarly, when former students report sexual abuse by teachers, they may realize through other students’ testimonies that they were not the only ones. Feeling isolated and guilty as a child and later in life goes back to the strategies of perpetrators. This is why the realization, often later in life, that they were not the only victim is a central moment in the process of bearing witness for the individual and for the socio-political dimensions of transitional justice.^8^

Bearing witness is also a matter of forms of generational transmission of knowledge, such as family stories passed on from older to younger family members, a knowledge that is usually changed by the process of transmission. It is also about unspoken matters that are nevertheless transmitted within families or other groups and leave an impression on children, for example.

The analysis of testimonies given to the German Inquiry focuses on the knowledge that can be produced through bearing witness to child sexual abuse in relationships of care and upbringing.

### Time and Reappraisal

The category of time is central to the reappraisal of sexual abuse in childhood and adolescence. Without an awareness and knowledge of the “entanglements” of past, present, and future, a critical examination of injustice is not possible. However, time must also be understood as a critical category. There are several reasons for this. During the work of an inquiry, time is closely linked to the concept of speed. Careful procedures slow down investigations into the past, and clarifications of data protection and rights around freedom of expression and privacy can also take many months. Many survivors are at an advanced age and wish to obtain information about their individual cases and possible support and redress without further delay.

The option of charging a perpetrator with the criminal offense of child sexual abuse is not available due to the statute of limitations. This means that, after a certain number of years have elapsed, a perpetrator, the person who has been accused, can no longer be prosecuted and offenses can no longer be investigated before the criminal courts. This was a central reason for the establishment of the Inquiry in Germany.

The subjective reappraisal process varies greatly. People who have testified to the Inquiry have been at different stages in their lives. But in public, survivors are confronted with the expectation that their pain and sorrow caused by the abuse must also have an end. This is where everyday myths come into play, such as the idea that time heals all wounds.

The category of time in its various dimensions is thus a basic condition for the reappraisal process and at the same time can prove to be a potential obstacle and a possible cause of failure.

Lifetime abuse refers to time, especially in relation to the individual’s life course (Easton & Kong, 2020). This means that survivors often describe when and how they were particularly confronted with memories of abuse in childhood and the consequences it had for them, such as mental health problems during phases of transition, for example, when moving from studying to employment or when becoming a mother. In the case of the testimonies of survivors of child sexual abuse, time becomes a central category and is directly or implicitly a subject of discussion. For the study, time was defined by the forms of past, present, and future. The survivors speak about their past childhood, but also about children in the present. They criticize the child protection of the past and make suggestions for future child protection measures.

Given the research focus, three research questions were formulated for the study: How do survivors and other witnesses address time? What knowledge about sexual abuse, its dynamics, and circumstances can be generated when the focus of analysis is on time? What themes in the testimonies are connected to aspects of lifetime abuse?

## Methods

### Sample

The sample of testimonies comes from the “story portal”^9^ on the German Inquiry’s website, with over 120 testimonies from confidential hearings and written reports. In a methodologically carefully designed project, the Inquiry prepared testimonies for publication on the website, always based on the survivor’s own words.^10^

The reports were selected via the online portal’s filter function. This function can be used to filter reports by different criteria, including 11 crime contexts; gender (female, male, or other); growing up in the FRG (1949 to the present) or the GDR (1949–1990); and decade.

The following filter functions were applied:

Context of crimes: Family, social environment, out-of-home care, church, leisure time, school, day-care center.
Gender: Female, male, other.
Decade: 1950s, 1960s, 1990s, 2000s.

**Figure 1.**
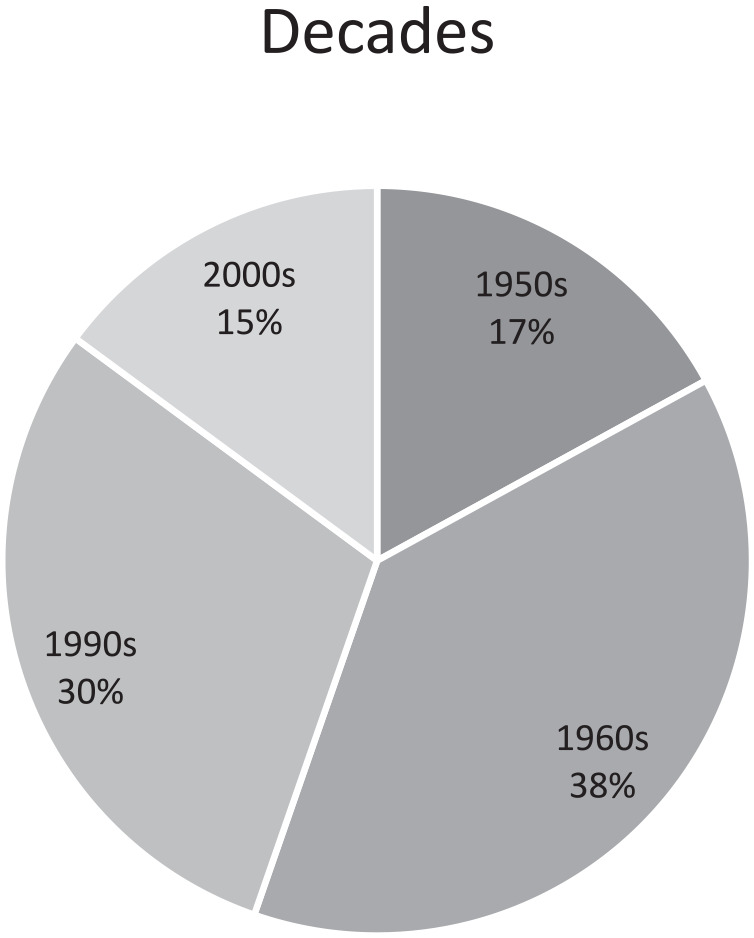
Decades of childhood.

Figure 1^11^ shows the distribution across the decades covered by this study.

In order to include contrasting reports, two decades further back in time (1950s and 1960s) and two more recent decades (1990s and 2000s) were chosen for the sample.

Through this narrowing-down process, 44 stories were identified. Only the reports from the 1950s and the 2000s were used for the in-depth analysis. This decision was also based on a contrastive sampling strategy.

The sample includes the following gender ratio: 66% female and 32% male witnesses. Even though it is now clear that boys can also be affected by child sexual abuse, the proportion of male witnesses appearing here is comparatively high.

**Figure 2.**
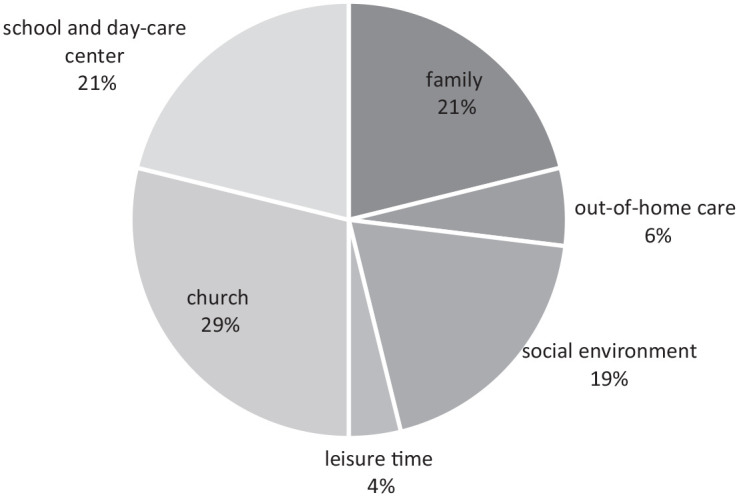
Context of crimes.

Figure 2^12^ shows the context of crimes and a comparatively smaller proportion of reports relating to family; overall this does not correspond to the proportions found in the total number of reports to the Inquiry and is due to the strategy of representing different contexts as broadly as possible through the online portal.^13^ Social environment as a context is usually also close to the family. The church as a context of crimes is broadly represented here with cases from the Catholic and Protestant Churches as well as other religious communities (Keupp et al., 2016; Dreßing et al., 2018; Kowalski, 2018).

### Structured Content Analysis

The structured content analysis approach, as described by Kuckartz (2016) was chosen for the analysis of the 44 testimonies (see Spraitz & Bowen 2016). In the first step of the analysis, each report was coded on the basis of categories developed using the three research questions. The reports were a maximum of three pages long. For the in-depth analysis of the research question about time, the testimonies relating to the 1950s and the 2000s were then selected to provide a possible contrast.

The reports were read line by line and analyzed deductively according to the following heuristic: Time as temporal location of the survivor. Important signal words are “earlier,” “back then,” etc., biographical times, time as a diagnosis of the present, understanding practices and processes associated with time, for example, processes of violence or abuse, time themes in concrete contexts, for example, family or court proceedings, crime progression, and perpetrator strategies.

In the second step, inductive coding was carried out in order to facilitate possible further thematization. The category “practice of waiting” was identified.

In the third analysis step, six reports of child sexual abuse from the 2000s and eight reports from the 1950s were analyzed in depth. Only the themes that were identified in both groups—childhood in the 1950s and childhood in the 2000s—are presented below.

## Results

Four overall themes of time could be distinguished: The identification of turning points, time to make sense of what happened, the practice of waiting, and time-bound experience of bearing witness.

### The Identification of Turning Points

Turning points are often used in accounts to describe the beginning of the sexual abuse and its scope, as well as to mark the decision to externalize and disclose the abuse. In cases where the start of the assaults is described as a turning point, this is framed by two narratives. First, the narrative of the loss of childhood is found in the reports. A survivor who was abused by a Catholic priest in the 1950s writes that the “happy child” died within her. For the second narrative, the beginning of the abuse is seen as a turning point toward a long series of traumatic events, often up to the point of testimony, that is, as a turning and starting point of lifetime abuse. Stories of loss are embedded in a temporal “before and after” structure.

Survivors from both groups refer to historical events and discourses in their accounts. Turning points toward speaking about what was experienced are mentioned both from childhood and from later phases of life. Especially in remembered childhood, such turning points are imbued with the hope of an end to the abuse and the hope that help will be forthcoming (Andresen, 2018).

The testimonies document the survivors’ experience that they were often not listened to and believed as a child and that this experience is repeated later in life. Against this backdrop, survivors in this sample emphasize the importance of public media discourse. It has obviously contributed to a turning point in their lives to deal with their abusive childhood again and to actively bear witness. This is exemplified by a quote from a survivor who suffered sexual violence at the hands of a Catholic priest:

After the recent frequent reporting in the media about sexual abuse in the church, I have dealt again with what I had repressed and made the decision to talk about my years of abuse. Today, everything has to be listened to and believed.

Knowledge transfer and school education can also lead to turning points. This is described by a survivor who was sexually abused by her maternal grandfather as a child in the 2000s and experienced sexual abuse from her father a few years later as an adolescent. She says that her school enabled her to recognize the injustice and encouraged her to disclose the abuse to her mother.

In fourth grade, we had sex education. That’s when I realized that what my grandpa was doing was not right and told my mother.

The survivor was helped, but that did not stop the second perpetrator from abusing her later, and in this case, she received no support.

The way survivors describe turning points indicates how decisions may also be prompted by an event or influenced by a media report. These testimonies were therefore about turning points initiated by the actions, perpetrators, and bystanders involved, as well as about turning points that encouraged the survivors to speak about, disclose, or reappraise their experiences. In the case of the fourth-grade girl, the turning point led to an end to the abuse by her grandfather, but not to permanent protection.

The thematization of turning points corresponds to the thematization of the painful processes of working through and coming to terms with the abuse, which are often described as long and drawn out. These turning points are followed by hard work in the process of personal reappraisal, as illustrated by the quote from a survivor who grew up in the 1950s. As a child, she had fled from what is now Masuria (East Prussia) and was then sexually abused by her stepfather.

In the meantime, I am now a happy grandmother. I am incredibly grateful for that. It was hard work to get this far and overcome the severe depression.

### Time to Make Sense of What Happened

Apart from time as a dimension in the development of the child, the concept of “time to make sense” is also about opportunities for education and acquisition of knowledge. However, the analyses of survivors’ accounts of family sexual abuse also indicate that it took a long time to understand and perhaps verbalize the injustice they experienced if they grew up from birth in a climate of fear, disregard, and violation. If a toxic environment is normal, children may not suspect or understand the injustice until, for example, they meet other families, such as those of their peers. The testimonies of survivors of child sexual abuse both in the 1950s and in the 2000s provide detailed insights into this.

The example of a survivor who suffered sexual violence from her high school teacher for many years from the age of 14 and was forced into an intense relationship of dependency illustrates the connection between the time needed to make sense of what happened and the support provided by good information. In her testimony, she describes her active search for words and terms, including researching the term “sexual abuse” on the internet. Knowledge certainly proves to be an important first step, but survivors then often need time to reach a place of recognition and acknowledgment.

This word “abuse,” it took me a while before I could acknowledge that in relation to myself. I started researching on the internet what “abuse” even means. Eventually, a few years ago, I asked a lawyer how it would be if I reported him. I wished to finally fight back. And I also felt a responsibility to see that such people were convicted. But in the end I decided not to spend another two years of my life doing it, because I had already sacrificed nine years.

Knowledge and expert advice in this case also led to the survivor being able to make an autonomous decision. It was also a decision about how she should spend her lifetime. In this case, she decided against pressing charges in order not to sacrifice any more years of her life to this perpetrator.

### The Practice of Waiting

With the evaluation focus on time, the practice of waiting has emerged as a phenomenon. Here, too, historical and biographical aspects intertwine, but above all, the structural conditions for investigation, support, and reappraisal become visible. Waiting illustrates how dependence and inequality affect the lives of victims. Many of them in the older and younger age group report long waiting times for a therapy place, compensation, or state aid funds. They wait for appointments, for assessments, and then for forensic reports to be prepared and forwarded. Waiting is a central experience across all offense contexts and is a theme in all age groups in the sample.

The sample studied includes one report from a recent witness, the mother of a girl who disclosed her experience of abuse to her mother when she was 4 years old. The mother describes how her daughter disclosed her experience while she was washing the child in the bathtub. The child found a way to describe which parts of her body the neighbor, a friend of the parents, had touched. The victim’s mother and father believed their child and, after counseling and time to think, began the long and drawn-out process of pressing charges. The girl had to make a statement, an event that already involved a long period of waiting.

The testimony happened three months after the incident and our child could still remember everything. Then it was back to waiting to see what would happen next, and we were still not allowed to say anything to the family [of the accused]. . .

Four months later, our neighbor’s house was finally searched. Now the stress really started. It triggered such hatred! A’s [the accused’s] wife couldn’t understand what was happening. Since it took months for any pictures from the mobile phone and laptop to be analyzed, we had to keep waiting. . .

When the prosecution filed charges, we were happy and thought, now things are moving forward. The first date for the trial in the fall was canceled three days before. Why? We didn’t know. We waited another three months until we were told a new date: In six months, which means two years after the crime!

This long excerpt from the testimony of a mother who is both a witness and an affected family member exemplifies the repeated powerlessness of survivors of sexual child abuse in the investigation, response, and finally criminal proceedings. Waiting proves to be a significant experience and a process to be endured, and it puts survivors in a position of dependence.

### Time-Bound Experience of Bearing Witness

Bearing witness is a practice, it takes place in time and space. Seeing the child of “then” and being located in “now” through writing indicates the nonlinear dynamics of past, present, and future (Andresen, 2018). As with the findings on turning points, the issue here is the contemporary context, as survivors describe their motivation to testify to the Inquiry, with the encouragement of the Inquiry and society’s concern for a critical investigation, and they look to the future. They are experienced experts who are in a position to speak representatively and as advocates for children today, because as in the case of one survivor from the 1950s, “Children won’t hold up a sign saying ‘Me too’ to draw attention to themselves.”

The reports also give concrete insights into the situation and practice of giving testimonies. What is described is the experience of time, the experience of reporting, and the feelings associated with it. The intertwining of time dimensions emerges significantly in these passages. A survivor, who was first in an infant home in the 1950s and then in a children’s home for many years, addresses those who read his written report to the Commission: “Even today, when I write this to you, tears come to my eyes.”

Especially in the written reports, different modes of envisioning and describing the situations in which testimony is given become apparent. The survivor’s own current life situation is presented in the light of the past and, in the sense of a balance sheet, future prognoses regarding the individual’s biography are sketched out. In addition, it is a reflection on the rights and protection of children today and in the future. This is done by distancing the individual from their abusive past.

## Discussion

The four themes that have been identified in which time plays a role refer to different aspects of lifetime abuse. It emerges that for those who testify to the Inquiry, the past is not completely left behind, but has an impact on their present and future prospects. Studies focusing on older people also point to this: If they experienced abuse in residential institutions as children, they often articulate their fear of being placed in care homes for senior citizens (Caspari et al., 2021).

### Turning Points

References to turning points frame the events of both childhood and the individual’s entire life course. In particular, this involves narratives about the beginning and end of violence. This is instructive in light of the fact that survivors of family sexual abuse in particular report how diffuse their sense of time was as a child because they were always expecting an assault (Andresen et al., 2021). Turning points for disclosure are associated with the public and media discussion about child sexual abuse. This has already been pointed out in other studies in which biographical and discourse analysis were intertwined (Pohling, 2021). However, it is also known from research that disclosure cannot be viewed as an individual event, as a single turning point, but instead must be understood as a process (Tener & Murphy, 2014).

The analysis revealed that the thematization of a lost childhood plays a major role for survivors. The images of childhood and the injustice toward children, who are particularly dependent, are aspects that are now being examined more intensively in research on transitional justice (Hamber & Lundy, 2020; Wright, 2017). In particular, this explores the idea that childhood should be as happy as possible and that children in particular should be given all the necessary protection. Since children have little control over those around them, child sexual abuse in the survivors’ reports is therefore seen as a great injustice in biographical retrospect.

The insight into the importance of turning points indicates a need for further research. In this analysis, turning points illustrate typical memories of significant moments from childhood or moments in the present, and they refer to the processual and thus to the course of time, to lifetime aspects that are involved in some way with the disclosure (Tener & Murphy, 2014). A turning point, however, also describes a moment at which it is possible for a person to address traumatic childhood experiences due to a changed social climate through truth-seeking initiatives (Sköld & Swain, 2015). This is where the importance of inquiries comes to the foreground, as they invite survivors to bear witness.

### Sense Making

A frequent theme in all testimonies to the Inquiry is how survivors of sexual abuse try to understand what is happening to them, why a person who is often close and really familiar to them causes fear, even mortal fear, disgust, fright, and helplessness (Andresen, 2018; IICSAG, 2019). The explanations of understanding as a child and/or adolescent and the understanding that comes in later phases of life certainly refer to development—cognitive, emotional, and social—and to limitations as well as potential at certain ages. The literature shows that it is very difficult for children and adolescents to understand what has happened to them when they have no knowledge whatsoever, when they have no vocabulary for sexual body parts, or when sexuality is a taboo subject, and this provides a starting point for today’s approaches to prevention and sex education (Jensen et al., 2005). Studies also show that a child’s first concern is always to understand. Then they look for the “right” time to approach a person to disclose their experience (Staller & Nelso-Gardell, 2005). In addition to the difficulty of understanding and actually finding the words, there is the fear of an even greater loss of control following disclosure (Attrash Najjar et al., 2022).

At the same time, however, it emerges that age is also a social category and that maturity does not automatically lead an individual to an understanding of their own history of violence and abuse. Critical events in the individual’s life course, understanding of and routes out of the dynamics of violence and abuse do not depend solely on individual age-related abilities, but also on the family environment, as well as on society’s openness to the concerns of survivors. The process of overcoming the silence helps them to disclose their experiences (Andresen et al., 2021; Briggs et al., 2023; IICSAG, 2019).

This is also evident in surveys of young people, as Maschke and Stecher (2018) show in their representative survey of pupils in grades 9 and 10. Here the results show that young people want to be asked and approached by capable adults in order to be able to trust someone. In the testimonies of adult survivors, it is often said that no adult around them asked the “right” questions (Andresen, 2023).

### Practice of Waiting

The theme of waiting contains a lifetime component that strikingly illustrates the persistent feelings of powerlessness that can accompany the experience of child sexual abuse at different stages of life. The long duration of criminal proceedings and family court proceedings are a major problem for children, at least in Europe. For this reason, attempts have been made in recent years to achieve a child-friendly justice system (Liefaard & Kilkelly, 2018). The aim is to shorten criminal investigations and proceedings involving children and young people and to reduce the burden on children as witnesses, as well as to enhance the reliability of children’s testimonies (Jol & Stommel, 2023; Poole et al., 2015). Overall, the results are also consistent with victim-centered approaches, regardless of age (Hamber & Lundy, 2020).

Having to wait has the same effect of putting people in the later stages of life in situations of dependence as it does with children. Survivors of child sexual abuse, especially older people, have had to wait a long time for society to address the phenomenon of child sexual abuse, enable reappraisal, and investigate claims for restitution and care (IICSAG, 2019). Many survivors have told the Inquiry about long waiting times, for example for a place in therapy or for decisions on applications to insurance companies. They wait in Germany for appointments for assessments and then for forensic reports to be prepared and forwarded.

The experiences and practices of waiting are therefore an important issue in understanding the consequences of child sexual abuse over a lifetime.

### Bearing Witness

The situation in which witnessing takes place is meaningful to the overall concept of bearing witness in light of transitional justice and child sexual abuse (Andresen, 2021; Gallen, 2023). How must a space be designed so that a confidential hearing is experienced as helpful and supportive (Hamber & Lundy, 2020)? What support do survivors need when writing a report or going to a confidential hearing so that the memory of the violent childhood does not overwhelm them in the here and now? These descriptions of the situations intertwine past, present, and future in the life course of individuals and on the societal level. Here, possibilities also arise in childhood theory to make space for the past from childhood (Hanson, 2017) and to come analytically close to what it means to experience sexual abuse in this specific phase of life.

The study has its limitations. These lie in the reports prepared for the online portal together with the survivors. This and the heuristic on time and temporality provide a first, rather exploratory insight into how knowledge about lifetime abuse can be gained through bearing witness. Further elaboration is needed on the nonlinear aspects that have become apparent.

## Conclusions

All four themes look at the experiences of survivors of child sexual abuse from a life course perspective. This also provides insights into lifetime abuse, in particular, the significance of turning points in the individual’s life course is important. Witnessing and analyzing testimonies is therefore a way of gaining knowledge and insight into lifetime abuse.

Research on child sexual abuse commissions and truth-telling initiatives have presented new knowledge about the extent of trauma experienced by survivors, some into old age (IICSAG, 2019). The study presented here, with its contrastive analysis of testimonies about child sexual abuse in the 1950s and 2000s, can hopefully provide new inspiration for research into lifetime abuse. In the future, the focus must be on what kind of traumatic childhood repetitions people fear in old age which therefore leads them not to seek support, for example, from the health and care system.

Bearing witness is also a kind of bridge to the former child (Andresen, 2018). It covers the past of the childhood experiences and recount, the present of the witnesses, and their perspectives on their personal future, but also the future of improved child protection and children’s rights and human rights (Liefaard & Kilkelly, 2018). Their own future and that of potential victims of child sexual abuse are strong motivations for survivors approaching the Inquiry and sharing their own stories (IICSAG, 2019).

After all, time as lifetime is a central factor in the work of the commissions of reappraisal. At what point in their lives and in what social climate do adults talk about child sexual abuse and other experiences of violence they have had? International comparative research on the commissions would be necessary to answer this question. The potential of the bearing witness approach for research into lifetime abuse should be investigated further in the future.

## Notes

1. Runder Tisch Sexueller Kindesmissbrauch (RTSKM). (2011). *Abschlussbericht*. Runder Tisch. Sexueller Kindesmissbrauch in Abhängigkeits- und Machtverhältnissen in privaten und öffentlichen Einrichtungen und im familiären Bereich. https://www.bmfsfj.de/resource/blob/93204/2a2c26eb1dd477abc63a6025bb1b24b9/abschlussbericht-runder-tisch-sexueller-kindesmissbrauch-data.pdf

2. Independent Commissioner for Child Sexual Abuse Issues in Germany. (2023). *Central portal for issues involving sexual violence against children and adolescents.* https://beauftragte-missbrauch.de/en/

3. German Survivors’ Board. (2023). *Survivors’ Board at the Independent Commissioner for Child Sexual Abuse Issues.* https://beauftragte-missbrauch.de/en/survivors-board/survivors-board-at-ubskm

4. Unabhängige Kommission zur Aufarbeitung sexuellen Kindesmissbrauchs. (2023). *Independent Inquiry into Child Sexual Abuse in Germany.* Aufarbeitungskommission. https://www.aufarbeitungskommission.de/english/

5. Nationaler Rat gegen sexuelle Gewalt an Kindern und Jugendlichen. (2023). *Forum für den Dialog zwischen Verantwortungsträger*innen aus Politik und Gesellschaft.* https://www.nationaler-rat.de/de/

6. The Independent Inquiry into Child Sexual Abuse. (2023). *IICSA for England and Wales.* https://www.iicsa.org.uk/

7. Unabhängige Kommission zur Aufarbeitung sexuellen Kindesmissbrauchs. (2023). *Definition und Begriffsbestimmungen Sexueller Kindesmissbrauch. Aufarbeitungskommission.* https://www.aufarbeitungskommission.de/kommission/aufarbeitung/sexueller-kindesmissbrauch/

8. Movie reference “Und wir sind nicht die Einzigen” by Christoph Röhl from 2011 about the disclosure of systematic abuses at the Odenwaldschule (boarding school) in 2010.

9. Geschichtenportal. (2023). *Geschichten, die zählen*. https://www.geschichten-die-zaehlen.de

10. Partly abridged and the final draft agreed with the survivor. Nevertheless, in the analysis account must be taken of the fact that these documents are edited.

11. Multiple responses possible.

12. Multiple responses possible.

13. See Endnote 9.

14. Poelchau, H. W., Briken, P., Wazlawik, M., Bauer, U., Fegert, J. M., & Kavemann, B. (2015). *Bonner Ethik-Erklärung. Empfehlungen für die Forschung zu sexueller Gewalt in pädagogischen Kontexten.* https://www.bmbf.de/bmbf/shareddocs/downloads/files/ethikerklaerung-1.pdf?__blob=publicationFile&v=2
